# Dynamic changes of monocytes-related immune activation in people with HIV switching to long-acting injectable cabotegravir plus rilpivirine

**DOI:** 10.1038/s41598-026-44013-6

**Published:** 2026-03-15

**Authors:** Maria Antonella Zingaropoli, Mariasilvia Guardiani, Anna Carraro, Eeva Tortellini, Federica Dominelli, Michele Antonacci, Cosmo Del Borgo, Raffaella Marocco, Giulia Mancarella, Lorenzo Ansaldo, Piergiorgio Pace, Livia Bresciani, Serena Vita, Fabio Mengoni, Roberta Campagna, Ombretta Turriziani, Maria Rosa Ciardi, Claudio Mastroianni Mastroianni, Miriam Lichtner

**Affiliations:** 1 Department of Public Health and Infectious Diseases, Sapienza University of Rome, Rome, Italy; 2 Microbiology and Virology Unit, University Hospital Policlinico Umberto I, Sapienza University of Rome, Rome, Italy; 3 Department of Neurosciences, Mental Health, and Sense Organs, Sapienza University of Rome, NESMOS, Rome, Italy; 4 Infectious Diseases Unit, Sapienza University of Rome, SM Goretti Hospital, Latina, Italy; 5 Department of Molecular Medicine, Sapienza University of Rome, Rome, Italy

**Keywords:** Long-acting therapy, Cabotegravir, Rilpivirine, Monocytes, Dendritic cells, HIV-DNA, flow-cytometry, SCD163, SCD14, Diseases, Immunology, Medical research, Microbiology

## Abstract

The aim of this study was to examine the dynamic changes in monocyte/macrophage- and dendritic cell (DC)-related immune activation in people with HIV (PWH) before switching to long-acting (LA) injectable cabotegravir (CAB) plus rilpivirine (RPV) (T0), after six (T6m) and twelve months (T12m). A total of 30 aviremic PWH switching to LA CAB plus RPV, and 32 healthy donors (HDs) were enrolled. At each time point, in comparison to HDs, PWH exhibited higher classical and intermediate monocyte counts and lower slanDC and pDC counts. Compared to HDs, in PWH non-classical monocyte counts were higher only at T0. PWH exhibited elevated sCD163 and sCD14 plasmatic levels at each time point, in comparison to HDs. No virological failure was observed. At T12m, no differences in the total cell-associated HIV-1 DNA levels compared to T0 were found. The longitudinal evaluation showed a significant reduction in non-classical and intermediate monocyte counts at T12m compared to T0 and an increase in classical monocyte and pDC counts. Furthermore, a reduction in sCD14 plasma levels was observed. The constant drug concentrations provided by CAB plus RPV injections appear to be effective for a sustained and deep virological suppression that may reduce monocyte/macrophage activation in PWH.

**Supplementary Information:**

The online version contains supplementary material available at 10.1038/s41598-026-44013-6.

## Introduction

Antiretroviral therapy (ART) provides durable viral suppression and can significantly prolong the life expectancy among people with human immunodeficiency virus (PWH)^1^. Current guideline-recommended first-line regimens require lifelong daily oral therapy that, although effective and well tolerated, can engender dissatisfaction, contribute to self-stigma, and increase the risk of nonadherence to treatment and treatment failure^2–4^.

There is a substantial interest among PWH for less frequent dosing options^5^. Consequently, ongoing therapeutic research has been directed at simplifying ART in order to improve adherence and satisfaction, and long-acting (LA) injectable regimens represent one of the alternatives^5–7^. LA injectable intramuscularly formulations, with a frequency of 8 weeks, has been developed for cabotegravir (CAB), which is an integrase strand-transfer inhibitor (INSTI), and rilpivirine (RPV), which is a nonnucleoside reverse-transcriptase inhibitor (NNRTI)^8,9^. The LATTE-2, ATLAS and FLAIR clinical trials have demonstrated the non-inferiority of LA injectable CAB plus RPV in maintaining virological suppression, compared to the oral counterpart in switching strategy^6,10^.

Despite these important achievements in the therapeutic field, HIV infection remains a chronic condition, characterized by a persistent inflammation and immune activation, which induces several complications that are currently identified as non-AIDS-related conditions^11–13^. The underlying mechanisms are not fully understood, but an important contribution derives from the increased production of inflammatory chemokines and mediators, and the permanent activation of the innate immune system, including the T-cells, natural killer (NK) cells, dendritic cells (DCs) and monocytes/macrophages^11,14–16^. Although HIV-RNA plasma determination remains the gold standard for monitoring virological response, accumulating evidence indicates that also total cell-associated HIV-DNA quantification is predictive of disease progression and poor immunological response during suppressive ART^17^.

Among circulating DCs that undergo alterations in number (plasmacytoid dendritic cells, pDCs), HLA-DR expression (myeloid dendritic cells, mDCs) or both parameters (slanDCs) during HIV infection, after the initiation of ART, an increase in absolute count (pDCs) and a decrease of HLA-DR expression on mDCs, slanDCs and non-classical monocytes was observed^16^. However, changes in the innate immune compartment and its activation markers, such as sCD163, a marker of monocyte/macrophage activation, and sCD14, a marker of immune activation and microbial translocation, are more variable^14,18^. Of note, some studies found that plasma levels of sCD163 remain elevated in PWH despite ART, confirming residual monocyte/macrophage activation after HIV suppression^19–21^.

This residual immune activation may represent a therapeutic target to improve the prognosis of PWH.

Furthermore, in the pre-LA therapy era, the analysis of circulating biomarkers improved the understanding of immune responses to HIV infection and showed their usefulness in predicting HIV-associated outcomes^14,22,23^.

Given the lack of real-world data on the impact of this novel approach on inflammation in natural immunity compartment, the aim of this study was to investigate the dynamic changes in monocyte/macrophage and DC subsets, their soluble activation markers and total cell-associated HIVDNA in PWH switching to LA injectable CAB plus RPV.

## Results

### Characteristics of the participants

**Fig. 1:**
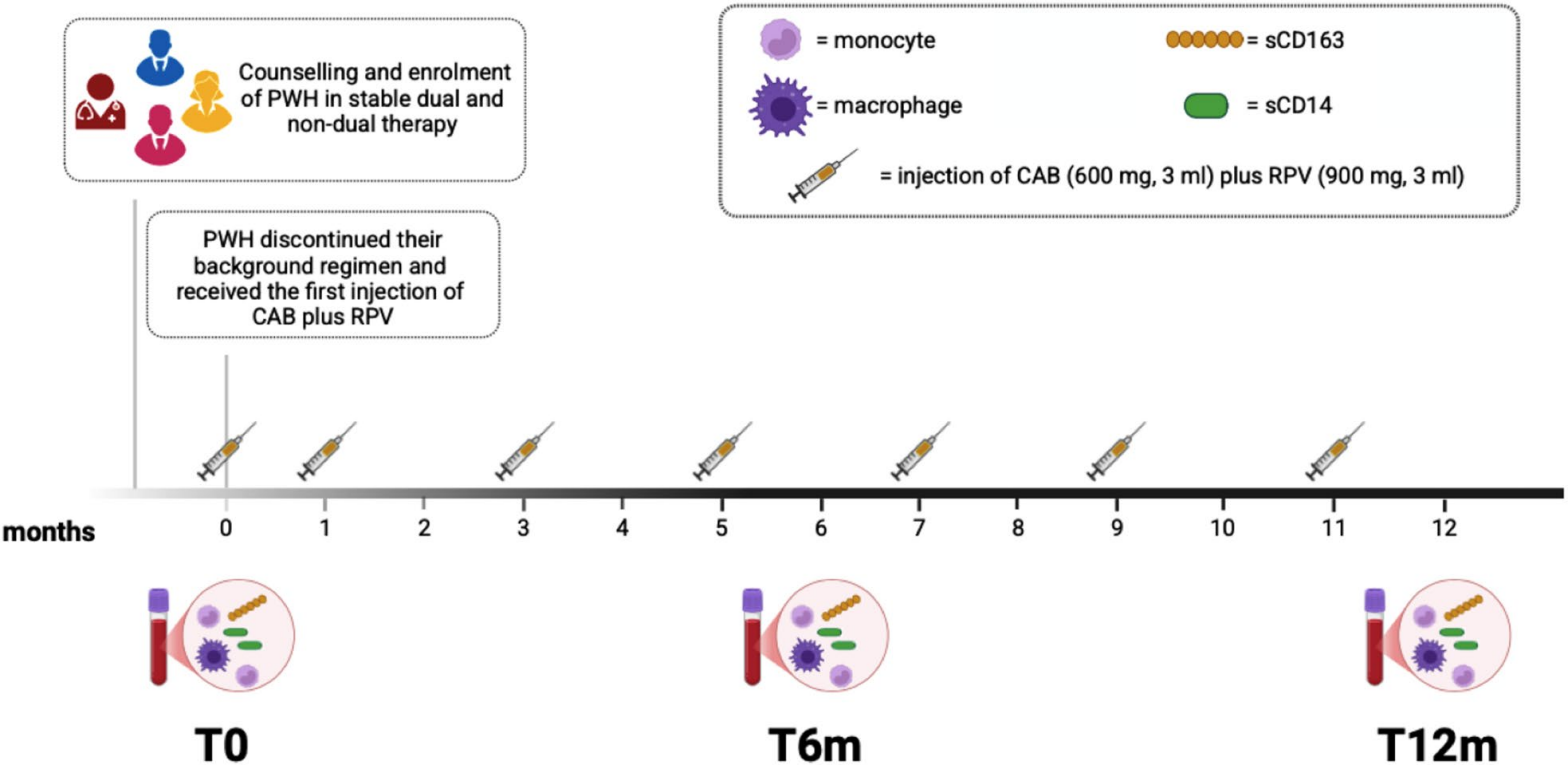
Study design. The number of peripheral blood monocyte/macrophage and dendritic cell (DC) subsets were assessed longitudinally, as well as monocyte/macrophage plasma activation markers. Furthermore, the quantification of both plasma HIV-1 RNA and total cell-associated HIV-1 DNA was performed too. Three time points were considered: before the start of LA injectable CAB plus RPV (T0), after six (T6m) and twelve months (T12m).

A total of 30 aviremic PWH discontinued their background regimen, switched to LA injectable CAB plus RPV, and were enrolled in the study (Fig. 1). The main demographic and clinical characteristics are listed in Table 1. All the enrolled PWH were ART-experienced (12 dual/18 triple therapy) and 100% had a viral load of < 50 copies/mL for at least 1 year, prior to initiating LA injectable CAB plus RPV. All PWH had at least one comorbidity, and 50% of them had more than one. The most prevalent comorbidities were endocrine and metabolic disorders (43%), followed by neuropsychiatric conditions (27%), cardiovascular diseases (23%), and dermatological disorders (23%). Further details on the distribution of other comorbidities are provided in Table 1. In addition, all PWH tested negative for CMV DNA; however, 90% had detectable levels of IgG anti-CMV.

Finally, 32 HDs matched for sex at birth and for age were included in the study.

**Table 1:** Demographic and clinical characteristics of study population. Data are shown as median (interquartile range, IQR). PWH: people with HIV, HDs: healthy donors, n: number, n.a.: not available.

age, years	PWH (*n* = 30)	HDs (*n* = 32)
	47 (35–56)	51 (41–55)
sex at birth (male/female)	21/9	22/10
body weight, kg	76 (67–86)	61 (56–73)
aspartate aminotransferase, AST, U/L	22 (19–27)	n.a.
alanine aminotransferase, ALT, U/L	18 (15–32)	n.a.
creatinine, mg/dL	0.95 (0.80–1.04)	n.a.
triglycerides, mg/dL	106 (74–138)	n.a.
high-density lipoprotein, HDL, mg/dL	55 (45–67)	n.a.
low-density lipoprotein, LDL, mg/dL	123 (105–141)	n.a.
total cholesterol, mg/dL	196 (180–222)	n.a.
one comorbidity, n (%)	30 (100%)	19 (60%)
more than one comorbidity, n (%)	15 (50%)	5 (16%)
endocrine/metabolic	13 (43%)	12 (38%)
neuropsychiatric (depression)	8 (27%)	0 (0%)
dermatological	7 (23%)	2 (6%)
cardiovascular	7 (23%)	10 (31%)
onco-hematological	4 (13%)	0 (0%)
pulmonary	4 (13%)	0 (0%)
renal	2 (7%)	0 (0%)
HBcAb positivity, n (%)	3 (10%)	0 (0%)
HCV Ab positivity, n (%)	0 (0%)	0 (0%)
anti-CMV IgG positivity, %	27 (90%)	30 (94%)
time from HIV diagnosis, years	14 (7–23)	n.a.
CD4 nadir cell count, cell/µl	246 (89–408)	n.a.
current CD4 cell count, cell/µl	705 (525–988)	n.a.
current CD8 cell count, cell/µl	688 (495–998)	n.a.
CD4/CD8 ratio	0.95 (0.69–1.50)	n.a.
HIV-RNA < 50 copies/ml, n (%)	30 (100%)	n.a.
dual/triple previous therapy, n	12/18	n.a.

### Monocyte/macrophage and DC subsets in study population

Monocytes were classified according to their CD14 and CD16 expression into three subsets: classical monocytes (CD14 + + CD16-), intermediate monocytes (CD14 + CD16+) and non-classical monocytes (CD14-CD16+). Finally, non-classical population was further characterized based on the expression of the 6-Sulfo LacNAc (slan) marker, identifying slan+ subpopulations (SlanDCs).

An analysis was conducted in which the absolute counts of monocyte/macrophage subsets in PWH and HDs at each time point were compared.

**Fig. 2:**
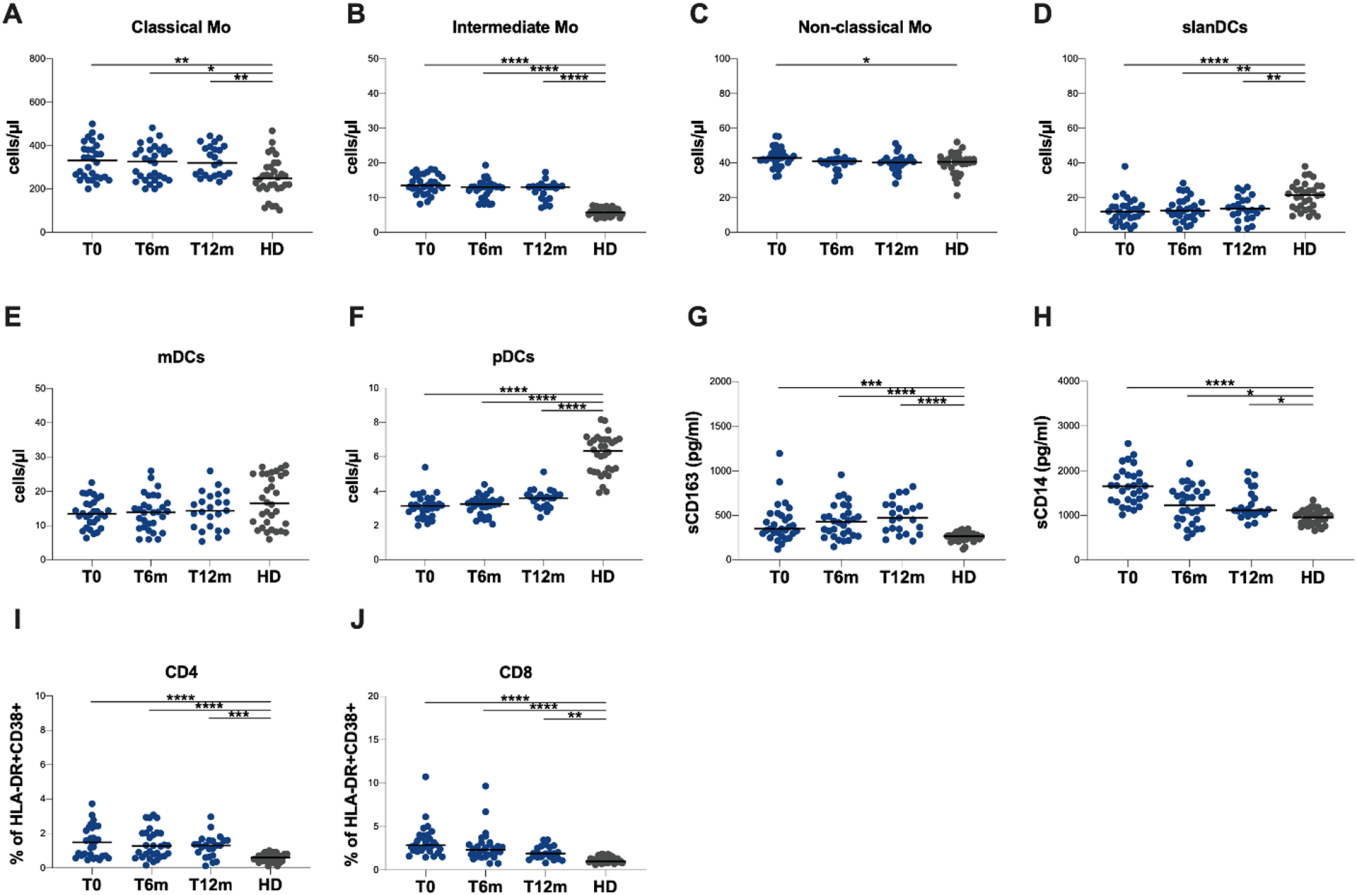
Absolute counts of monocyte and dendritic cell subsets and their plasma activation markers in study population. **(A)** Classical monocyte (CD14 + + CD16−) absolute counts/µL blood, **(B)** intermediate monocyte CD14 + CD16+ absolute counts/µL blood, **(C)** non-classical monocyte (CD14 + CD16±) absolute counts/µL blood, **(D)** slanDC absolute counts/µL blood, **(E)** mDC absolute counts/µL blood, **(F)** pDC absolute counts/µL blood, **(G)** plasma sCD14 levels, **(H)** plasma sCD163 levels in PLWH and HD. Mo: monocytes, T0: before starting LA injectable CAB plus RPV, T6m: six months following LA injectable CAB plus RPV, T12m: 12 months following LA injectable CAB plus RPV, PWH: people with HIV, HD: healthy donors, mDCs: myeloid dendritic cells, pDCs: plasmacytoid dendritic cells. Dunn’s post-test p is represented above the horizontal line connecting the compared groups. Horizontal bars represent median values. Kruskal-Wallis tests and descriptive statistics can be found in Table 2. *p < 0.05, **p < 0.01, ***p < 0.001, ****p < 0.0001.

At each designed time point, the absolute counts of classical and intermediate monocytes were found to be significantly higher in PWH than in HDs (T0: *p* = 0.0009 and *p* < 0.0001, respectively; T6m: *p* = 0.0061 and *p* < 0.0001, respectively; T12m: *p* = 0.0005 and *p* < 0.0001, respectively) (Table 2; Fig. 2A and B, respectively). The absolute counts of non-classical monocytes exhibited a higher value compared to HDs only when compared with those observed in PWH at T0 (*p* = 0.0035) (Table 2; Fig. 2C). No significant differences were observed in the absolute counts of non-classical monocytes in PWH at both T6m and T12m when compared with those of HDs (Table 2; Fig. 2C).

As reported in Table 2; Fig. 2D, the absolute counts of slanDCs were lower in PWH than HDs at each time point (T0: *p* = 0.0012; T6m: *p* = 0.0104; T12m: *p* = 0.0414). In PWH, at each time points, no significant differences were observed in the absolute counts of mDCs when compared with those of HDs (Table 2; Fig. 2E). At each time point the absolute counts of pDCs were lower in PWH than HDs (T0: *p* < 0.0001; T6m: *p* < 0.0001; T12m: *p* < 0.0001) (Table 2; Fig. 2F).

Finally, in comparison to HDs, at T0 PWH exhibited higher percentages of intermediate monocytes (*p* = 0.0004), and non-classical monocytes (*p* < 0.0001). Furthermore, higher percentages of non-classical monocytes were observed at T12m compared HDs (*p* = 0.0037) (Table 2, Supplementary Fig. 2).

**Table 2:** Evaluation of the monocyte and dendritic cell subsets and their plasma activation markers in study population. Data are shown as median (interquartile range, IQR). PWH: people with HIV, n: number, HDs: healthy donors.

	PWH (*n* = 30)			HDs (*n* = 32)
	T0	T6m	T12m	
absolute count				
classical monocytes (cell/µl)	331 (254–389)	326 (240–380)	321 (265–395)	229 (201–300)
intermediate monocytes (cell/µl)	13.5 (12.0–16.8.0.8)	13.1 (10.9–14.0)	13.1 (9.8–13.3)	5.8 (5.2–7.0.2.0)
non-classical monocytes (cell/µl)	42.9 (41.2–46.8)	41.0 (39.8–41.5)	40.2 (37.3–42.8)	40.4 (34.0–42.1.0.1)
slanDCs (cell/µl)	11.9 (7.8–15.4)	12.4 (8.9–17.7)	13.7 (8.4–18.7)	20.6 (12.7–26.0)
myeloid dendritic cells, mDCs (cell/µl)	3.2 (2.6–3.6)	3.3 (2.6–3.5)	3.6 (3.1–3.8)	6.3 (5.2–7.0.2.0)
plasmacytoid dendritic cells, pDCs (cell/µl)	13.5 (10.6–17.1)	13.9 (9.5–17.2)	14.4 (9.6–18.6)	14.2 (8.6–25.2)
percentages				
classical monocytes	57.5 (51.8–66.2)	58.6 (49.7–65.6)	53.4 (46.7–66.3)	52.3 (40.5–62.8)
intermediate monocytes	1.7 (1.5–1.9)	1.6 (1.4–1.8)	1.6 (1.3–1.9)	1.2 (1.1–1.7)
non-classical monocytes	2.4 (1.9–2.7)	2.1 (1.7–2.4)	1.7 (1.3–2.2)	1.3 (1.1–1.5)
slanDCs	1.7 (1.2–2.3)	1.8 (1.3–2.6)	1.7 (1.4–2.6)	1.7 (1.1–2.6)
myeloid dendritic cells, mDCs	40.6 (28.3–52.7)	39.0 (25.5–49.8)	34.3 (29.0–49.2.0.2)	43.6 (35.3–53.2)
plasmacytoid dendritic cells, pDCs	43.5 (31.9–56.4)	40.2 (30.8–53.4)	40.0 (33.5–56.3)	37.8 (31.3–42.6)
soluble CD14, sCD14 (ng/ml)	1650 (1333–1912)	1222 (884–1540)	1111 (1025–1479)	956 (805–1100)
soluble CD163, sCD163 (ng/ml)	348 (290–503)	427 (272–547)	472 (333–621)	264 (231–291)

The investigation of the absolute counts of monocyte subsets in PWH according to previous ART at each time point revealed no differences between dual and triple therapy (Supplementary Fig. 1).

In comparison to HDs, PWH exhibited elevated plasma levels of sCD163 (T0: *p* = 0.0005; T6m: *p* < 0.0001; T12m: *p* < 0.0001) and sCD14 (T0: *p* < 0.0001; T6m: *p* = 0.0127; T12m: *p* = 0.0105) at each time point (Table 2; Fig. 2G and H, respectively). The investigation of the plasma level of sCD163 and sCD14 in PWH according to previous ART at each time point revealed no differences between dual and triple therapy (Supplementary Fig. 1).

**Fig. 3:**
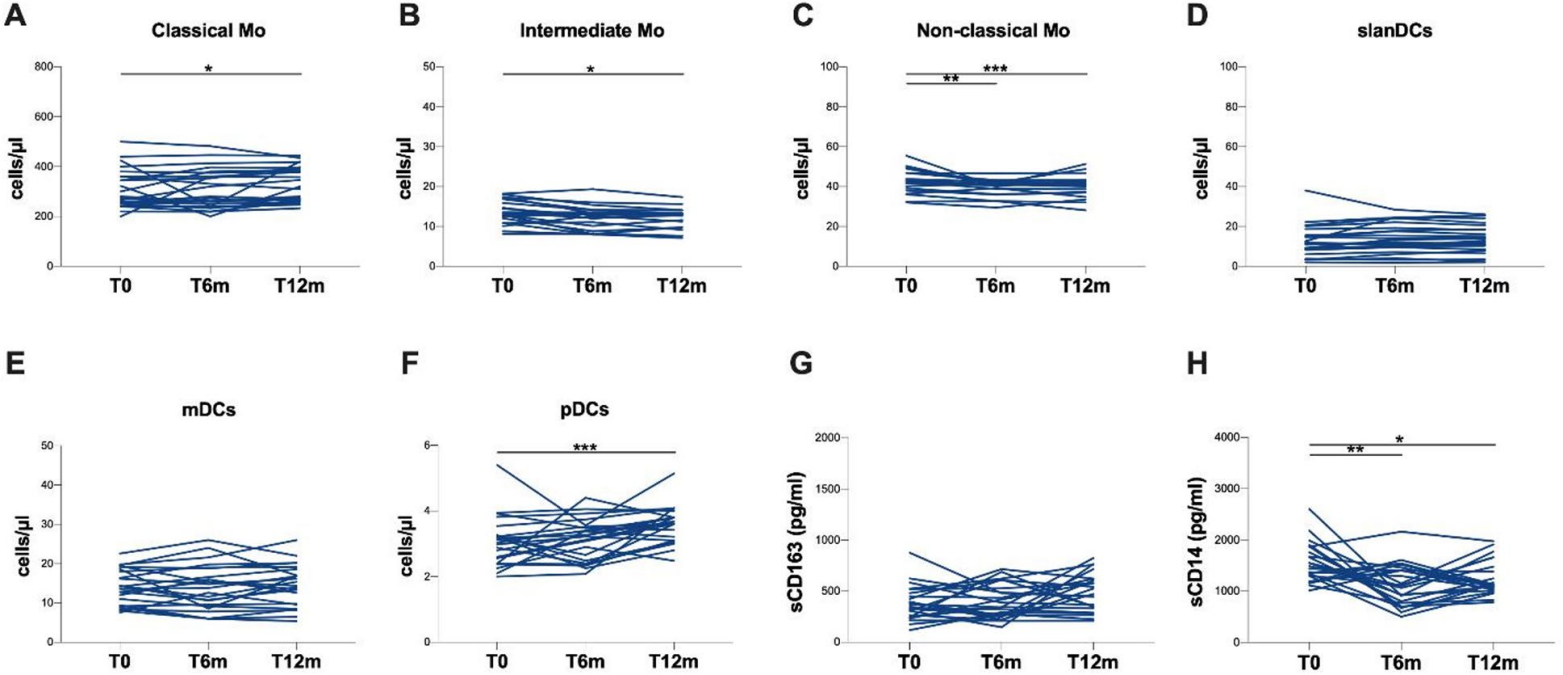
Longitudinal evaluation in study population. **(A)** Classical monocyte (CD14 + + CD16−) absolute counts/µL blood, **(B)** intermediate monocyte CD14 + CD16+ absolute counts/µL blood, **(C)** non-classical monocyte (CD14 + CD16+) absolute counts/µL blood, **(D)** slanDC absolute counts/µL blood, **(E)** mDC absolute counts/µL blood, **(F)** pDC absolute counts/µL blood, **(G)** sCD163 plasma levels, **(H)** sCD14 plasma levels in PLWH. Mo: monocytes, T0: before starting LA injectable CAB plus RPV, T6m: six months following LA injectable CAB plus RPV, T12m: 12 months following LA injectable CAB plus RPV, PWH: people with HIV, mDCs: myeloid dendritic cells, pDCs: plasmacytoid dendritic cells. Descriptive statistics can be found in Supplementary Table 1. *p < 0.05, **p < 0.01, ***p < 0.001.

Among 23 PWH, the longitudinal evaluation of the absolute counts of monocyte subsets revealed a statistically significant reduction in the number of intermediate monocytes at T12m in comparison to T0 (*p* = 0.0102) (Table 3; Fig. 3B). Similarly, a statistically significant reduction in the number of non-classical monocytes at both T6m and T12m were observed compared to T0 (*p* = 0.0014 and *p* = 0.0005, respectively) (Table 3; Fig. 3C). Conversely, a statistically significant increase in the number of classical monocytes at T12m in comparison to T0 was observed (*p* = 0.0300) (Table 3; Fig. 3A).

A statistically significant increase in the number of pDCs at T12m in comparison to T0 was observed (*p* = 0.0008) (Table 2; Fig. 3F). No statistically significant differences were observed in the absolute counts of slanDCs and mDCs (Table 3; Fig. 3D and E, respectively).

Finally, a significant reduction in plasma levels of sCD14 at both T6m and T12m compared to T0 was observed (*p* = 0.0039 and *p* = 0.0159, respectively) (Table 3; Fig. 3H). No statistically significant differences in the plasma levels of sCD163 were observed (Table 3; Fig. 3G).

**Table 3:** Longitudinal evaluation of absolute counts monocyte and dendritic cell (DCs) subsets and monocyte/macrophage plasma activation markers in study population. Data are shown as median (interquartile range, IQR). PWH: people with HIV, n: number.

classical monocytes (cell/µl)	PWH (*n* = 23)		
	280 (251–360)	280 (240–375)	321 (265–395)
intermediate monocytes (cell/µl)	13.1 (12.0–15.9.0.9)	12.9 (10.2–13.5)	13.1 (9.8–13.3)
non-classical monocytes (cell/µl)	42.6 (39.2–46.8)	40.9 (30.8–41.4)	40.2 (37.3–40.8)
slanDCs (cell/µl)	11.8 (8.2–15.6)	13.4 (9.6–19.2)	13.7 (8.4–18.7)
myeloid dendritic cells, mDCs (cell/µl)	14.2 (9.4–18.6)	13.8 (9.2–18.8)	14.4 (9.6–18.6)
plasmacytoid dendritic cells, pDCs (cell/µl)	3.0 (2.4–3.3)	3.3 (2.5–3.5)	3.6 (3.1–3.8)
soluble CD14, sCD14 (ng/ml)	1488 (1299–1864)	1100 (768–1408)	1111 (1025–1479)
soluble CD163, sCD163 (ng/ml)	347 (248–481)	344 (261–528)	472 (333–621)

### HIV-1 RNA and total cell-associate HIV-1 DNA quantification

**Fig. 4:**
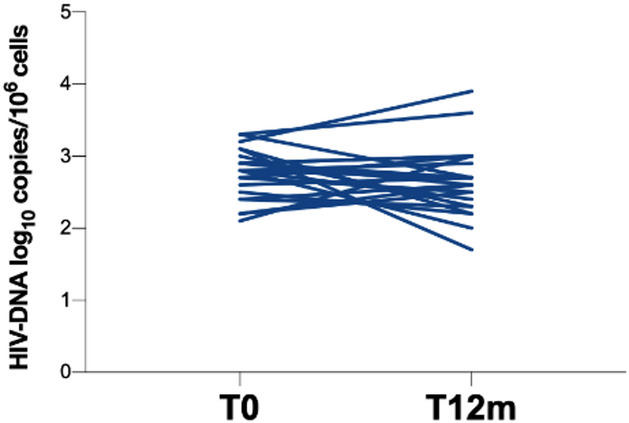
Quantification of total cellular HIV-1 DNA in peripheral blood mononuclear cells (PBMCs) of enrolled PWH. T0: before starting LA injectable CAB plus RPV, T12m: 12 months following LA injectable CAB plus RPV, PWH: people with HIV. Longitudinal evaluation was conducted using the non-parametric Friedman test.

For all enrolled PWH, at each time point the evaluation of HIV-1 RNA on plasma samples was performed. At both T6m and T12m, no virological failure was observed. As reported in Fig. 4, the total cell-associated HIV-1 DNA quantification performed at T0 and T12m showed no significant differences (2.8 [2.5–3.0.5.0] and 2.7 [2.3–3.0.3.0] log_10_ copies/10^6^ cells, respectively) (Fig. 4). No correlations between the total cell-associated HIV-1 DNA level and immunological parameters were found.

### Correlations with sCD14 plasmatic levels

**Fig. 5:**
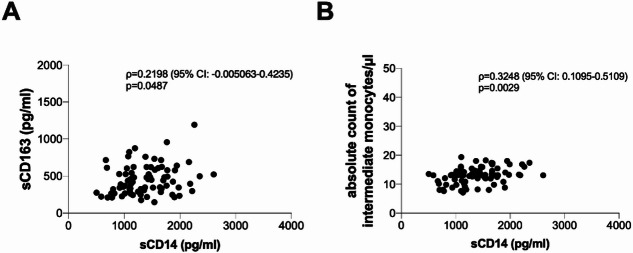
Correlations. (**A**) Positive correlation between plasmatic levels of sCD14 and sCD163. Linear correlation was evaluated by using the regression test, R^2^ = 0.05499 *p* = 0.0351. (**B**) Positive correlation between plasmatic levels of sCD14 and the absolute count of intermediate monocytes. Linear correlation was evaluated by using the regression test, R^2^ = 0.1325 *p* = 0.0008. CI: confidence interval.

Considering all PWH and all time points, a positive correlation between sCD14 and sCD163 plasmatic levels (ρ = 0.2198, *p* = 0.0487) was found as well as between sCD14 plasmatic level and the absolute count of intermediate monocytes (ρ = 0.3248, *p* = 0.0029) (Fig. 5A and B, respectively). No correlation between sCD14 and the absolute count of classical and non-classical monocytes were found as well as with the absolute count of pDCs, mDCs and slanDCs.

Investigating the relationship between sCD14 and sCD163 plasmatic levels at each time points, the positive correlation was observed only at T12m (ρ = 0.3858, *p* = 0.0352), whereas no correlations were observed at T0 and T6m (Supplementary Fig. 3). Furthermore, investigating the relationship between sCD14 plasmatic level and the absolute count of intermediate monocytes at each time points, the positive correlation was observed only at T0 (ρ = 0.2676, *p* = 0.0405), whereas no correlations were observed at T6m and T12m (Supplementary Fig. 3).

## Discussion

The present study investigated the impact of switching to LA injectable CAB plus RPV after 12 months on the differentiation and activation phenotype of peripheral monocyte/macrophage and DC subsets in PWH on a long, effective and stable ART.

The first finding is that nevertheless a prolong and good viro-immunological condition, our cohort of PWH on oral ART exhibited a persistent alteration in natural immunity, characterized by increased activated monocyte subsets and related soluble factors, along with a reduced number of pDCs and slanDCs. After 12 months of switching to LA injectable CAB plus RPV, there is a significant reduction in the absolute count of activated monocyte cells as well as in the plasma sCD14 levels and a recovery in the number of pDCs. In addition, no changes were detected in the quantitative levels of total cell-associated HIV-1 DNA.

To the best of our knowledge this is the first report describing the monocyte/macrophage and DC subsets along with their related activation markers and total cell-associated HIV DNA in PWH switching to LA injectable CAB plus RPV in the real world.

Conventionally, ART comprises three or more drugs with activity against HIV and is highly effective in diminishing the degree of immune activation^24^. It is important to note that, over the years, questions have been raised regarding the possibility of achieving virological suppression with a reduced number of antiretroviral drugs. This is an intriguing question, given that ART should be used for life and there is a possibility that its use could also induce cardiovascular and bone disease^25,26^. Consequently, the equilibrium between ART-induced toxicity and immune activation-related comorbidity is delicate. However, simplified regimens for the treatment of HIV-1 infection may improve adherence, side effects, and quality of life.

The HIV infection is characterized by a complex activated state of T-cells and monocytes, which react differently to different ART^16,27–29^. Whilst most of the research conducted on HIV-induced immunoactivation has historically focused on the role of CD8 + T-cells, there has been a growing interest in the potential involvement of monocytes and DCs in non-infectious complications observed among PWH. Although ART suppresses the over-secretion of cytokines in a non-selective manner, only the combination of RT inhibitors and HIV protease inhibitors has been demonstrated to reduce the monocyte subset expressing high levels of CD16^28,30,31^. Consequently, the effect of ART on the CD16high monocyte subset was selective. A decrease in the percentage of CD16high monocytes was observed, which was with a marked diminution of plasma viral load^28^. However, although it has been demonstrated that ART suppresses viral replication and reduces monocyte activation, it is acknowledged that some monocyte dysfunctions may persist, particularly in the latter stages of HIV infection^27^. Indeed, the balance between monocyte subsets is disrupted following HIV infection and, even PWH with long-term ART, the number and function of monocytes are not fully restored^32^. Furthermore, monocytes can facilitate the HIV reservoir and persistent viral infection^33^ as well as affecting the initiation and extension of immune activation and persistent inflammatory events^34^. These data are consistent with those of the present study, in which stable on long-term ART PWH showed persistent alterations in monocyte/macrophage subsets and higher plasma levels of sCD14 and sCD163 before switching to LA injectable CAB plus RPV, compared to the control group. Specifically, in the present study, PWH exhibited higher absolute counts and percentages of intermediate and non-classical monocytes before switching to LA injectable CAB plus RPV compared to the control group. This emphasizes the persistent nature of monocyte dysfunction and the potential reduced ability of monocytes to process and present antigens to T-cells^35,36^, even with the long-term effective ART treatment.

As discussed in several previous studies, it is imperative to maintain low levels of activation or inflammation biomarkers in order to achieve a favorable outcome for PWH undergoing ART^37–39^. In the present study, irrespective of the regimen prior to switching to CAB plus RPV, no significant alterations were observed in the absolute counts of monocyte/macrophage and DC subsets. Moreover, no substantial alterations were detected in the plasma levels of monocyte/macrophage activation markers. These findings are consistent with the results of several previous study which demonstrated that inflammatory biomarkers remained unchanged following the switching from a triple to a dual regimen^40,41^.

In the present study, a decline in the levels of inflammation/activation markers was observed 12 months after switching to LA injectable CAB plus RPV. Specifically, the data showed a decrease in the plasma levels of sCD14, a marker of monocyte activation and response to lipopolysaccharide (LPS), that has been associated with clinical and subclinical atherosclerosis^42^ and mortality^23^ during HIV infection^43^. Interestingly, our longitudinal analysis revealed two distinct kinetic patterns: while most patients showed a steady decline, a subset exhibited intermittent fluctuations before reaching stabilization. This heterogeneity may reflect individual differences in the immune-inflammatory “set-point”. Such transient increases could be attributed to pharmacokinetic variability during the initial phase of the switch; specifically, drug trough levels may fluctuate before achieving a definitive steady-state, potentially allowing for low-level, episodic monocyte activation. Furthermore, since sCD14 is a hallmark of microbial translocation, these fluctuations might represent transient “flares” of gut-derived inflammation in patients with pre-existing intestinal barrier dysfunction. Finally, despite undetectable CMV DNA, the high CMV-IgG seroprevalence (90%) in our cohort further suggests that subclinical immune triggers could contribute to sporadic sCD14 shedding during the therapeutic transition.

In addition, a positive correlation was found between sCD14 plasma levels and the absolute count of intermediate monocytes before starting LA injectable CAB plus RPV. This finding reflects the dynamics of the therapeutic switch. At T0, PWH exhibited a stable chronic inflammatory “set-point” where monocyte subsets and their activation markers (sCD14) were closely synchronized. Following the switch to LA injectable CAB plus RPV, a progressive decline in both parameters was observed although in different rates. Consequently, the present study suggests that LA injectable CAB plus RPV may regulate the excessive production of cytokines by the inflammatory monocytes, thereby reducing the levels of immune activation and inflammation^28^.

Although the reservoir role of monocytes is controversial, HIV-1 proviral DNA and replication-competent virus have been isolated in monocytes from ART-treated PWH with undetectable viral load^44–46^, suggesting that quantitative determination of HIV-1 DNA load might provide an useful measure of reservoir consistency and a functional parameter for monitoring therapy. In line with the finding of other authors^47–49^, in the present study no correlations between total cell-associated HIV-1 DNA and activation markers were found. This confirms that inflammation is not directly linked to the size of the HIV reservoir and highlights the role of other factors in maintaining high levels of immune activation in stable ART PWH. Furthermore, despite the blood represents a highly accessible sample, it is not a truly representative medium for measuring the latent reservoir in the entire body. Blood cells may or may not reflect other bodily tissues^50^.

The persistent alteration of monocytes activation is indicated by the unremitting plasma levels of sCD163 and the unalerted absolute count of intermediate monocytes. High-level monocyte activation could be due in part to coinfection such as HCV and CMV^20,51^. Specifically, in the present study, the plasma levels of sCD163 were found to be significantly higher in the study group when compared to the control group, thus indicating ongoing low-level monocyte activation^20^. CD163 is a hemoglobin-haptoglobin scavenger acceptor that has only been identified on monocytes and macrophages^52,53^. The expression of CD163 is greater on intermediate monocytes^32,54^. In the event of monocytes being stimulated by LPS, or FcγR cross-linking or oxidative stress happens, CD163 is known to be shed as sCD163. This process serves to decrease inflammation, cell stimulation and cell factor release, but is a marker of ongoing activation^20,55^.

Furthermore, several studies demonstrated an alteration of DC subsets during HIV infection^15,56^. Specifically, a severe reduction of pDC strictly linked to viral replication was reported and this reduction has been associated to clinical progression and virological failure under ART. However, these immunological parameters were highly influenced by therapy^16^. In line with previous data^16^, in the present study after LA injectable CAB plus RPV a significant increase of absolute count of pDCs, that may be linked to a reduction of residual HIV replication in tissue and reservoir.

Even if the present study apports, in our opinion, novel insight on this subject, presents several limitations.

Firstly, the observation period is relatively brief. Nevertheless, regarding viremia, which has been demonstrated to exert a substantial influence on inflammation, it has been observed that more than 85% of virological failures in three pivotal clinical trials transpired within a period of eight months^9,57,58^. This observation is in alignment with the time frame of this study.

Another limitation of this study is its relatively small sample size and the absence of a group including subjects with HIV undergoing continuous oral therapy. Furthermore, the investigation focused exclusively on total peripheral cell-associated HIV-1 DNA, with no differentiation made between intact and defective HIV-1 DNA.

In conclusion, although several functions of specific and innate immunity appear to be recovered during ART, some monocyte/DC dysfunctions persist. The significant reduction in inflammation and peripheral immune activation of the monocyte compartment observed after 12 months of LA CAB plus RPV underlines that LA injectable CAB plus RPV can exert an additive and beneficial effect probably due to specific pharmacokinetic and tissue penetration. The constant drug concentrations provided by directly observed CAB plus RPV injections appear to be effective in sustaining virological suppression and may be also effective in ameliorating innate immunity such as increasing pDC counts and reducing monocyte/macrophage activation in PWH. Indeed, the observed improvements after 12 months of LA CAB + RPV treatment may be attributed to several factors inherent to the long-acting formulation. Unlike daily oral ART, which is subject to 24-hour pharmacokinetic peaks and troughs, LA injectables provide constant drug exposure with a remarkably stable steady-state. This “flat” pharmacokinetic profile may more effectively suppress low-level, residual viral replication in sanctuary sites, such as the gut-associated lymphoid tissue, thereby reducing the chronic stimulus for monocyte activation and subsequent sCD14 shedding. Additionally, the high tissue penetration of both cabotegravir and rilpivirine in lymphoid compartments may directly counteract the local inflammation that characterizes microbial translocation (linked to sCD14) and DC dysfunction. Finally, the elimination of daily pill-taking may reduce the “psychological stress” of HIV management, which, although indirect, has been suggested to influence neuro-endocrine-immune pathways and systemic activation markers.

This kind of studies should be prioritized due to actual wide and increasing use of the LA delivery system for HIV therapy and prevention.

### Methods

#### Study design and population

This single-center prospective, observational study included PWH who were stable on ART under routine follow-up at the Infectious Diseases Unit of S.M. Goretti Hospital of Latina, Italy, and switched from a daily oral regimen to the LA injectable CAB plus RPV one, following the actual guidelines indications.

As reported in Fig. 1, at the time of enrollment, participants discontinued their background regimen, without oral lead-in and received the first injection, followed by injections administered after one month and then every two months for maintenance. Intramuscular ventrogluteal injections were administered by clinic nursing staff during dedicated visits within the hospital outpatient environment. PWH were provided with an injection planner for 12 months, which included all injection dates, information on post-injection pain management, and contact details for the team in case of any questions or concerns.

During routine clinical testing, peripheral whole blood samples were collected at the following time points: before starting LA injectable CAB plus RPV (T0), after six months (T6m) and after twelve months (T12m), along with demographic, epidemiological, clinical, and laboratory characteristics of all enrolled PWH (Fig. 1).

The absolute counts of peripheral blood monocyte/macrophage and DC subsets were then assessed on the collected blood samples. Absolute cell counts (cells/µl) were performed using a single-platform volumetric approach on a MACSQuant Analyzer (Miltenyi Biotec). This instrument utilizes a calibrated, high-precision syringe pump to measure the exact volume of the sample aspirated, allowing for direct absolute quantification without the need for counting beads. Briefly, 50 µl of whole blood was stained with monoclonal antibodies, followed by red blood cell lysis, reaching a final volume of 250 µl. For each sample, the entire final volume was aspirated and analyzed. The absolute count of each subset was then calculated by dividing the total number of events by the initial volume of whole blood, effectively accounting for the 5-fold dilution during sample preparation. This method ensured accurate and normalized counting across all specimens with a volumetric precision of ± 5%.

An evaluation of monocyte/macrophage plasma activation markers was conducted. Furthermore, the quantification of both plasma HIV-1 RNA and total cell-associated HIV-1 DNA was performed. At each designed time point, a comparison was done with a control group comprising age and sex-matched healthy donors (HDs). Finally, a longitudinal evaluation was performed for a subgroup of PWH whom had a complete with three time points.

The exclusion criteria for the enrollment in the study comprised coinfection with Hepatitis B virus (HBV), the presence of resistance to INSTIs and/or NNRTIs, and age < 18 years.

### Flow cytometry characterization of peripheral blood monocytes/macrophages, DCs and T-cells

As previously reported^25,59^, circulating monocyte/macrophage and DC subsets were characterized by multiparametric flow cytometry. Whole blood samples were collected in ethylenediaminetetraacetic acid (EDTA) tubes for this purpose. In summary, Brilliant Violet 510-conjugated anti-CD3, APC-conjugated anti-CD14, PerCp/Cy5.5-conjugated anti-HLA-DR, and PE/Cy7-conjugated anti-CD16 antibodies were used to identify specific monocyte subsets, namely non-classical monocytes (CD14 + CD16+), intermediate monocytes (CD14 + + CD16+) and classical monocytes (CD14 + + CD16-). The FITC-conjugated anti-M-DC8 and PE-conjugated anti-CD11c antibodies were used to identify slanDCs (M-DC8 + CD11c+). Finally, the Pacific Blue-conjugated anti-CD123 and PE-conjugated anti-CD11c antibodies were used for the identification of DC subsets, namely mDCs (CD123-CD11c+) and pDCs (CD123 + CD11c-). All the antibodies were from BioLegend, with the exception of M-DC8 and CD11c, which were from Miltenyi Biotec.

Briefly, 50 µl of whole blood were stained with the appropriate mix of monoclonal antibodies and incubated in darkness at 4 °C for 20 min. Subsequent to the initial incubation, red blood cells were lysed in the darkness at room temperature for 10 min using the BD Lyse solution (BD Biosciences). The cells were then washed in phosphate-buffered saline (PBS) containing 1% of Fetal Calf Serum (FCS). Finally, the samples were fixed in PBS containing 0.5% of formaldehyde (Sigma-Aldrich) prior to analysis. The stained samples were acquired using the MACSQuant Flow Cytometer (Miltenyi Biotec, Germany) and analyzed using FlowJo™ v10.6.2 software.

### Evaluation of sCD163 and sCD14 plasmatic levels

The quantification of sCD163 and sCD14 plasma levels was conducted on the collected samples in EDTA tubes using the Simple Plex™ Ella Assay (ProteinSimple, San Jose, CA, USA) on the Ella™ microfluidic system (Bio-Techne, Minneapolis, MN, USA) in accordance with the manufacturers’ instructions. As previously described^59^, plasma was obtained after centrifugation and immediately stored at − 80 °C until use. Prior to analysis, the Ella™ was calibrated using the in-cartridge factory standard curve. The limit of detection for sCD163 and sCD14 was determined to be 318 pg/ml and 1.0 pg/ml, respectively. The limits of detection were calculated by adding three standard deviations to the mean background signal determined from multiple runs.

### Plasma HIV-1 RNA and total cell-associated HIV-1 DNA quantification

For each time points, the HIV-1 RNA quantification assay was performed according to the manufacturer’s instructions, with a plasma input volume of 0.6 ml. The fully automated Alinity m platform features a single analyzer capable of extracting, amplifying, and detecting HIV-1. The dynamic range of quantification is 20 to 10,000,000 copies/mL (range, 1.30–7.00.30.00 logarithm to base 10 [log_10_] copies/mL).

The HIV-1 DNA quantification assay was performed at T0 and T12 on peripheral blood mononuclear cells (PBMCs) obtained from EDTA whole blood via Ficoll Histopaque (Amersham Biosciences, Sweden) density gradient centrifugation. As previously described^60^, the number of viable leukocytes was determined by trypan blue exclusion. PBMCs were stored at − 80 °C until use. Using the QIAamp^®^ Blood Mini Kit (Qiagen, Germany), genomic DNA was isolated, and the nucleic acid was subsequently quantified using the Qubit dsDNA HS Assay on a Qubit 4.0 fluorometer (Thermo Fisher, USA). Total cell-associated HIV-1 DNA, including integrated and al unintegrated forms, was determined using the Generic HIV-DNA Cell assay (Biocentric, France) following the manufacturer’s instructions. Total cell-associated HIV-1 DNA was expressed as log_10_ copies/10^6^ cells.

### Statistical analysis

All statistical analyses were conducted using GraphPad Prism v.9 software. A p-value of ≤ 0.05 was designated as statistically significant. The values are expressed as the median and the interquartile range (IQR). The non-parametric comparative Kruskal-Wallis test with Dunn’s post-test was used for comparing medians of PWH at each time point with those of HDs. Longitudinal evaluations were conducted using the non-parametric Friedman test with Dunn’s post-test was used for comparing medians of T0 with T6m and T12m. The association between sCD14 and sCD163 levels was assessed using a linear mixed-effects model to account for repeated measures per subject. Subject ID was entered as a random effect, while sCD14 was treated as a fixed effect.

## Supplementary Information

Below is the link to the electronic supplementary material.

Supplementary Material 1

## Data Availability

The data that support the findings of this study are available from the corresponding author, upon reasonable request.
