# Supplementary material for: Dynamic changes of monocytes-related immune activation in people with HIV switching to long-acting injectable cabotegravir plus rilpivirine
Source: Sci Rep. 2026 Mar 15;16:13580. doi: 10.1038/s41598-026-44013-6 (PMC13121530; doi:10.1038/s41598-026-44013-6)

**Supplementary Figure 1**. **Absolute count of** **monocyte and DC subsets and plasma activation markers in PWH stratified according to previous ART. A) Classical monocyte (CD14++CD16−) absolute counts/µL blood, B) intermediate monocyte CD14+CD16+ absolute counts/µL blood, C) non-classical monocyte (CD14+CD16+) absolute counts/µL blood, D) slanDC absolute counts/µL blood, E) mDC absolute counts/µL blood, F) pDC absolute counts/µL blood, G) sCD163 plasma levels, H) sCD14 plasma levels.** T0: before starting LA injectable CAB plus RPV, T6m: six months following LA injectable CAB plus RPV, T12m: 12 months following LA injectable CAB plus RPV, PWH: people with HIV, mDCs: myeloid dendritic cells, pDCs: plasmacytoid dendritic cells.


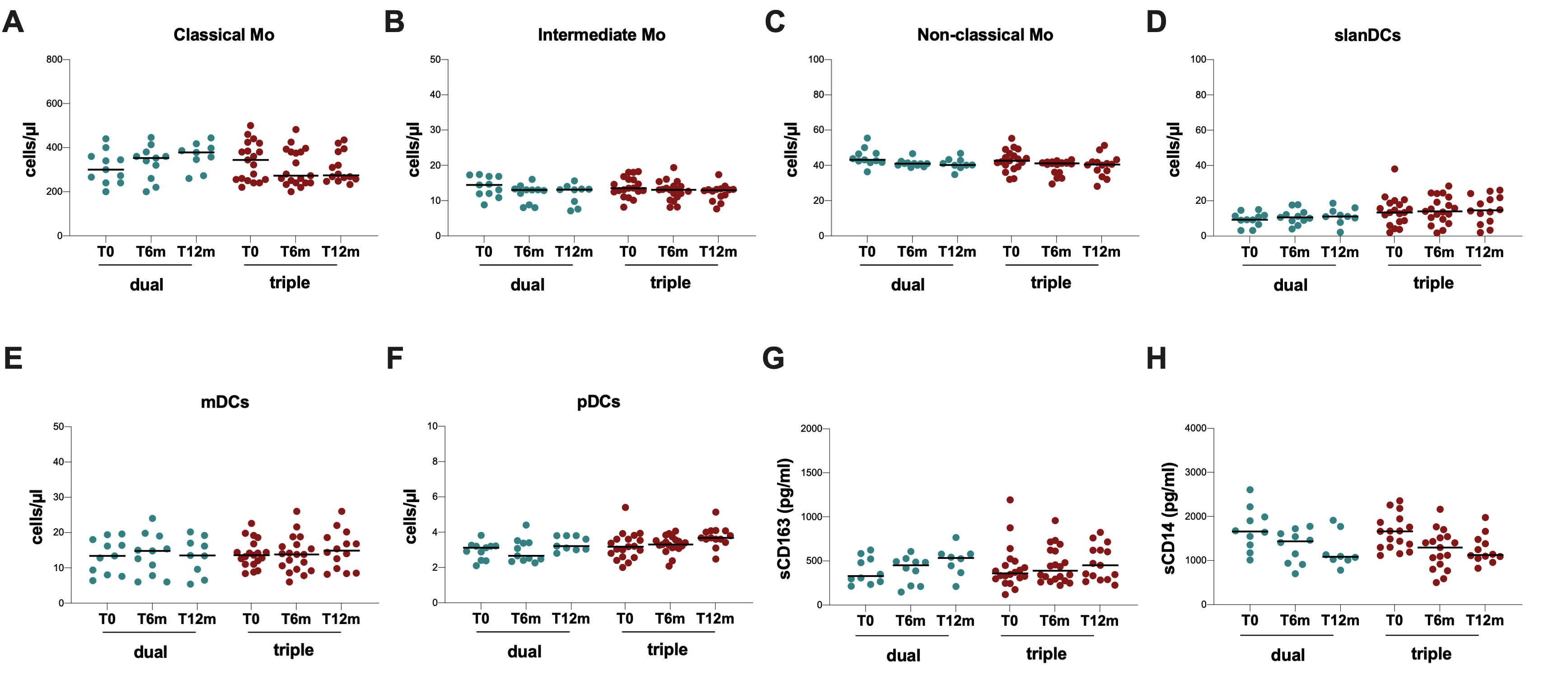


**Supplementary Figure 2. Percentages of monocyte/macrophage and DC subsets in the study population. A) Classical monocyte (CD14++CD16−) percentages, B) intermediate monocyte CD14+CD16+ percentages, C) non-classical monocyte (CD14+CD16+) percentages, D) slanDC percentages, E) myeloid dendritic cell (mDC) percentages, F) plasmacytoid dendritic cell (pDC) percentages in PLWH and HD.** T0: before starting CAB/RPV LA, T6m: six months following CAB/RPV LA, T12m: 12 months following CAB/RPV LA, PLWH: people living with HIV, HD: healthy donors. Dunn’s post-test p is represented above the horizontal line connecting the compared groups. Horizontal bars represent median values. Kruskal-Wallis tests and descriptive statistics can be found in Table 2. *p<0.05, **p<0.01, ***p<0.001, ****p<0.0001.


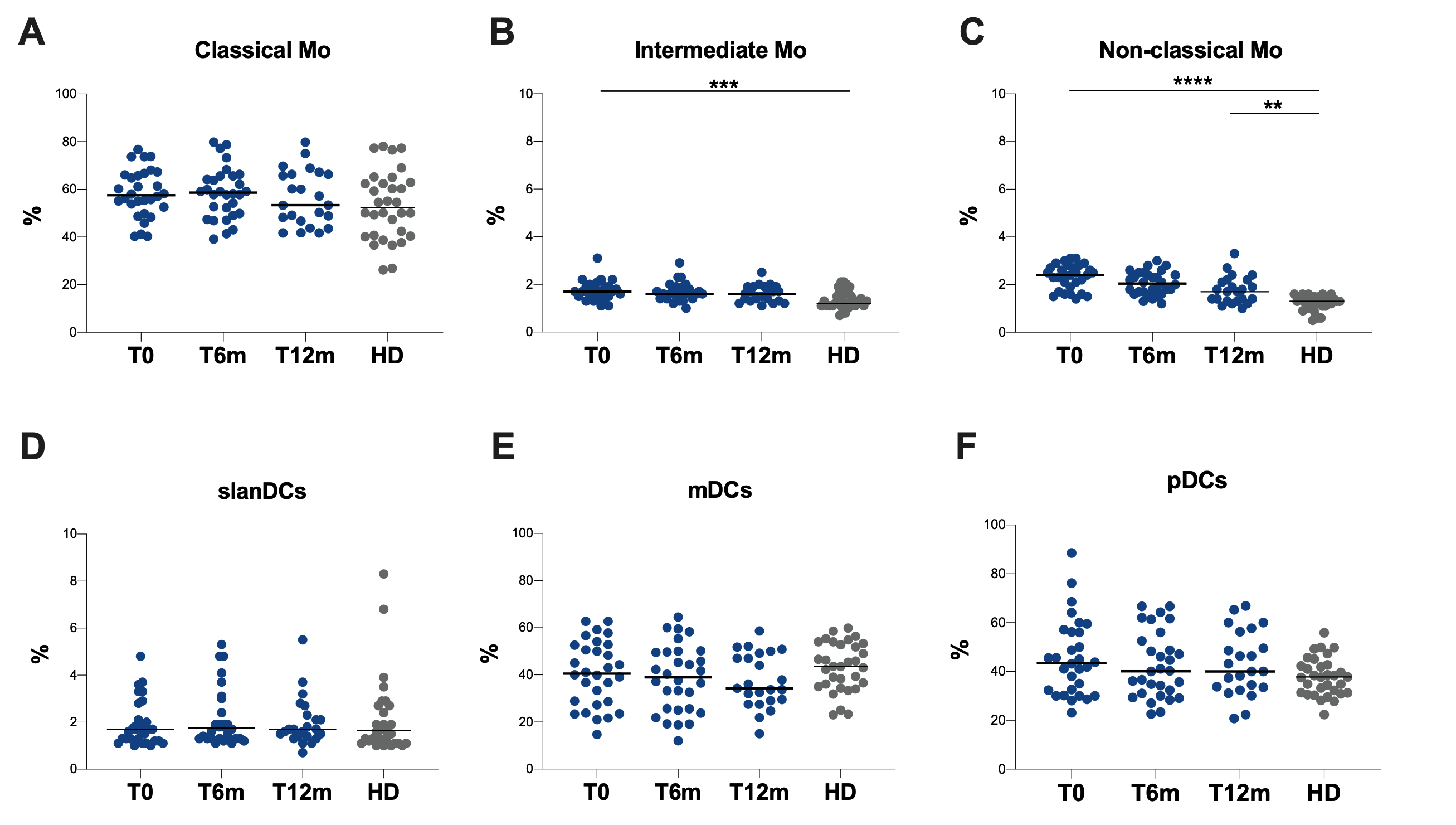


**Supplementary Figure 3. Correlations at each time point. A) Positive correlation between plasmatic levels of sCD14 and sCD163 at T0, B) at T6m, C) at T12m. D) Positive correlation between plasmatic levels of sCD14 and the absolute count of intermediate monocytes, E) T6m, F) T12m.** T0: before starting CAB/RPV LA, T6m: six months following CAB/RPV LA, T12m: 12 months following CAB/RPV LA, CI: confidence interval.


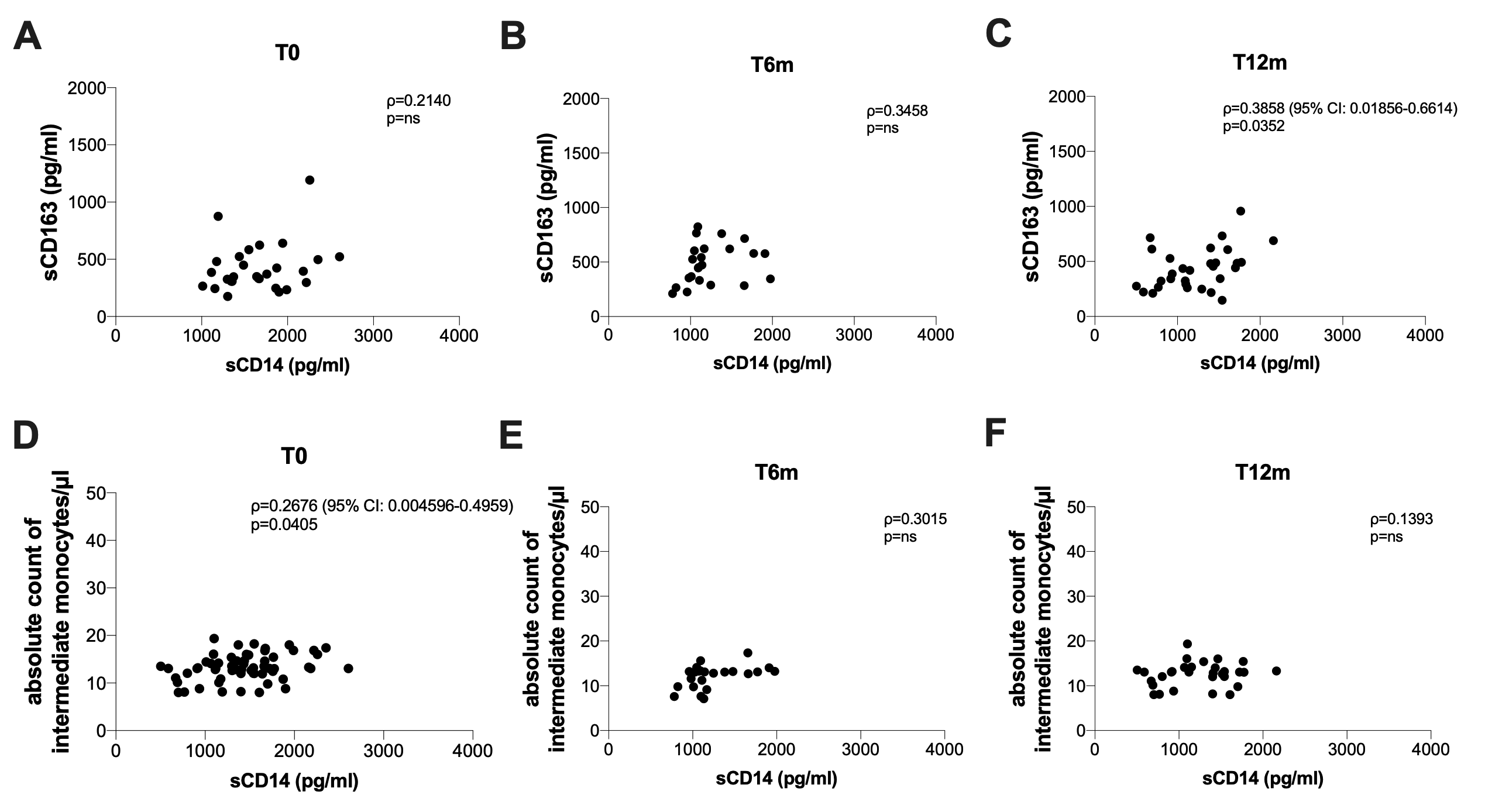

Supplement: Supplementary file 1 — Supplementary Material 1 [file 41598_2026_44013_MOESM1_ESM.docx]
